# Acceptance and Use of eHealth in Support and Psychological Therapy for People With Intellectual Disabilities: Two Cross-Sectional Studies of Health Care Professionals

**DOI:** 10.2196/52788

**Published:** 2024-11-12

**Authors:** Cathelijn Oudshoorn, Noud Frielink, Heleen Riper, Petri Embregts

**Affiliations:** 1 Stichting ASVZ Sliedrecht Netherlands; 2 Tranzo, Tilburg School of Social and Behavioral Sciences Tilburg University Tilburg Netherlands; 3 Amsterdam Public Health Mental Health program Department of Clinical, Neuro-& Developmental Psychology Vrije Universiteit Amsterdam Netherlands

**Keywords:** acceptance, health care professionals, intellectual disabilities, eHealth, disability, psychological therapy, support, cross-sectional survey

## Abstract

**Background:**

Acceptance of health care professionals is of paramount importance for the uptake and implementation of eHealth. The Unified Theory of Acceptance and Use of Technology (UTAUT) model is a widely used framework for studying health care professionals’ acceptance and actual use of eHealth among general client populations. However, there is limited understanding of the eHealth acceptance of health care professionals working with people with intellectual disabilities (ID).

**Objective:**

This study aimed to explore the applicability of the UTAUT model toward understanding the acceptance, intention to use, and actual use of eHealth among support staff and therapists working with people with ID.

**Methods:**

A total of 2 cross-sectional survey studies were conducted among health care professionals from 5 health care organizations for people with ID in the Netherlands in 2018 (n=311) and in 2021 during the COVID-19 pandemic (n=326). In addition to confirmatory and exploratory factor analyses to evaluate both the original UTAUT model and an extended version, descriptive analysis was used to explore participants’ characteristics, acceptance levels, and eHealth usage. Moderator analysis and multiple regression analysis were also used.

**Results:**

A confirmatory factor analysis indicated a poor fit for both the original 4-factor UTAUT model and the extended version. An exploratory factor analysis was then conducted, resulting in a more satisfactory 5-factor model after removing 1 item with a factor loading <.40. Internal consistency of the 5 factors ranged from acceptable to good (Cronbach α=.76-.85). Collectively, all factors predicted the intention to use eHealth in 2018 (*R*^2^=0.47; *F*_5,305_=54.885; *P*<.001) and in 2021 (*R*^2^=0.43; *F*_5,320_=49.32; *P*<.001). Participants scored moderately on all 5 acceptance factors in both 2018 and 2021. Moderator analysis indicated that age and voluntariness influence the relationship between factors that determined acceptance and intention to use eHealth.

**Conclusions:**

The findings from 2 cross-sectional studies conducted in 2018 and 2021, using an extended UTAUT model, gave a deeper understanding of eHealth acceptance among health care professionals who work with people with ID.

## Introduction

### Background

Health care organizations are increasingly incorporating eHealth, a term denoting the use of technology for promoting health, well-being, and health care [1]. This approach has also been adopted to provide support and psychological therapy to people with intellectual disabilities (ID). People with ID are characterized by significant limitations in intellectual functioning and adaptive behavior, encompassing conceptual, social, and practical adaptive skills [2]. The primary objective of professional support and psychological therapy offered by health care organizations for people with ID is to bridge the gap between individual capabilities and environmental demands [2,3]. These services are delivered in various settings, including residential and community-care environments. Given the lifelong and life-broad support required by people with ID, professional support plays a crucial role. In recent years, support and therapy have been increasingly delivered by using digital technology [4,5]. The use of eHealth in health care organizations serving people with ID, as in other health care sectors, accelerated during the COVID-19 pandemic in 2020 [6].

While some studies report positive experiences of health care professionals using eHealth for ongoing support or psychological therapy during the pandemic [7,8], others identify challenges in effectively delivering digital mental health support among people with ID [6,9,10]. A particular concern for health care professionals is building a working alliance virtually [11], which is crucial for the perceived value of eHealth usage. Factors such as digital literacy, availability of suitable equipment, and on-site support from direct support staff or relatives for people with ID to use equipment properly also affect the willingness of health care professionals to use eHealth [12,13].

Acceptance is likewise key in influencing health care professionals’ willingness toward eHealth [14], in terms of their perception of eHealth as appropriate, feasible, and suitable for delivering support or therapy [15,16]. Acceptance at an individual level is associated with the intention to use eHealth and contributes to the success or failure of eHealth implementation [17,18]. A commonly used theoretical model to explain the acceptance and usage (or nonusage) of eHealth in clinical practice is the Unified Theory of Acceptance and Use of Technology (UTAUT) [19].

### The UTAUT Model: The Role of Acceptance

The UTAUT model is a 4-factor model that aggregates various theories to explain individuals’ acceptance and usage of technology [19]. While initially designed for industry and business services [20], the model has also been applied in various health care contexts, such as rehabilitation [21], mental health counseling in family practices, psychotherapy, and pediatric care [22-24].

According to the UTAUT model, 3 factors—performance expectancy, effort expectancy, and social influence—are related to the behavioral intention to use eHealth, subsequently impacting the actual usage of eHealth. Performance expectancy refers to health care professionals’ perceived added value of eHealth, while effort expectancy represents the ease of becoming familiar with using eHealth applications. Social influence encompasses the perceived social pressure or support of important others, such as colleagues or managers, in relation to the intention to use eHealth. The fourth factor in the UTAUT model is facilitating conditions, which directly affect the actual usage of eHealth. Facilitating conditions relate to the extent to which health care professionals perceive the organizational context and available technological infrastructure as supportive of eHealth usage [19].

**Figure 1:**
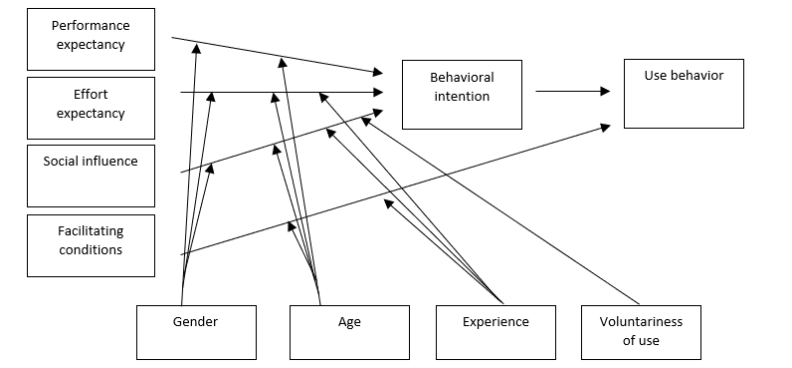
Original Unified Theory of Acceptance and Use of Technology (UTAUT) model (Venkatesh et al). Figure used with permission.

The correlations between performance expectancy, effort expectancy, and social influence with the behavioral intention to use eHealth can be influenced by 4 moderators: gender, age, experience, and voluntariness of eHealth use (more details in Figure 1 [19]). The moderator experience refers to the extent to which individuals feel comfortable and proficient using technology in daily life; the moderator voluntariness pertains to the degree of choice individuals have in using eHealth instead of being required to do so by the health care organization. The moderators age and experience may influence the correlations between facilitating conditions and use behavior.

### Acceptance of eHealth Among Health Care Professionals Working in Health Care Organizations for People With ID

Also, among health care professionals working with people with ID, both organizational and individual factors have been identified as influencing the acceptance and use of eHealth. Organizational factors include the culture surrounding information and communication technology implementation, technical challenges, and the quality of training received, which can impact the acceptance and use of eHealth either positively or negatively [25,26]. At the individual level, the digital experience and communication skills of both people with ID and support staff or therapists have been identified as facilitators or barriers to the acceptance and use of eHealth [5,8]. The willingness of support staff to introduce eHealth to people with ID and their belief in its potential benefits are also crucial toward fostering eHealth acceptance. Finally, several studies show that health care professionals’ digital experience can influence their acceptance and behavioral intention to use eHealth in practice [27-29].

While the aforementioned studies have identified relevant factors related to the organization, health care professionals, and clients for implementing eHealth in the care and support for individuals with ID, there is a lack of research that specifically addresses the acceptance and usage of eHealth among health care professionals working with this population using a theoretical model as starting point. Therefore, this study aimed to explore the applicability of the UTAUT model toward understanding the acceptance, intention to use, and actual use of eHealth among support staff and therapists working with people with ID. The study also explored the level of acceptance and use of eHealth for support and psychological therapy among support staff and therapists in the care and support for people with ID and examined whether the acceptance and usage of eHealth changed during the COVID-19 pandemic. The research questions derived from these aims are:

1. Is the UTAUT model applicable for understanding health care professionals’ acceptance and intention to use eHealth for support and psychological therapy among people with ID?
2. What is the level of acceptance and use of eHealth among support staff and therapists providing support and psychological therapy for people with ID, and did the acceptance and usage change during the COVID-19 pandemic?

## Methods

### Design

To investigate the acceptance and usage of eHealth among health care professionals in support and psychological therapy for people with ID, 2 cross-sectional, web-based surveys were conducted. The first survey took place in 2018, and the second survey, which included the same items as the 2018 survey, along with additional questions concerning COVID-19 and perceptions of working alliance when using eHealth, was administered in 2021. During this period, official measures included the conclusion of a lockdown period (from November 2020 to June 2021), the discontinuation of the 1.5-m social distancing measure in August 2021, the availability of vaccines, and a gradual reopening of society (eg, higher education resumed live classes) as indicated in the Central Government’s coronavirus timeline [30]. These 2 cross-sectional, web-based surveys resulted in 2 independent datasets.

### Participants

The participants in the study were support staff and therapists, including psychologists and experience-based therapists (eg, art or drama therapists), who used at least 1 eHealth tool (eg, secure videoconferencing tool or a mindfulness app) to support or provide psychological therapy to people with ID. The analysis in the 2018 survey included data from 311 eHealth users of 673 participating support staff and therapists. In the 2021 survey, data from 326 eHealth users were available.

The demographic characteristics of support staff and therapists who participated in the web-based survey in 2018 and 2021 are presented in Table 1. Participants were asked about their voluntary or mandatory use of eHealth within their health care organization in both years, with the majority reporting voluntary use (232/311, 74.6% in 2018 and 223/326, 68.4% in 2021). Only a small percentage of participants reported being obligated to use eHealth (52/311, 16.7% in 2018 and 54/326, 16.6% in 2021). A minority of participants (26/311, 8.4% in 2018 and 13/326, 4% in 2021) indicated that their organization had no specific policy regarding the use of eHealth. Both surveys also examined participants’ eHealth education and training. In the 2018 survey, 61.4% (191/311) reported not having received any education or training. Similarly, in the 2021 survey, 65.3% (213/326) of the participants reported a lack of education or training, and 74.2% (242/326) reported not having received any education or training within the past year.

**Table 1:** Participant characteristics of the web-based questionnaires in 2018 (n=311) and 2021 (n=326).

Demographics and attribute			2018 survey (n=311), n (%)		2021 survey (n=326), n (%)
**Gender**					
	Male	45 (14.5)		44 (13.5)	
	Female	266 (85.5)		281 (86.2)	
	Other	0 (0)		1 (0.3)	
**Age group (years)**					
	<30	54 (17.4)		67 (20.5)	
	30-39	113 (36.3)		103 (31.6)	
	40-49	73 (23.5)		78 (23.9)	
	≥50	71 (22.8)		76 (23.3)	
	Missing	0 (0)		2 (0.6)	
**Education**					
	Lower	1 (0.3)		1 (0.3)	
	Secondary	92 (29.6)		114 (35)	
	Higher	218 (70.1)		204 (62.6)	
	Other	0 (0)		5 (1.5)	
	Missing	0 (0)		2 (0.6)	
**Profession**					
	Support staff	243 (78.1)		232 (71.2)	
	Psychologist	56 (18)		83 (25.5)	
	Experience-based therapist	12 (3.9)		11 (3.4)	
**Work domain**					
	Community care	53 (17)		67 (20.6)	
	Residential care^a^	158 (50.8)		175 (53.7)	
	Daycare center	35 (11.3)		31 (9.5)	
	Expert center	46 (14.8)		41 (12.6)	
	More than one	16 (5.1)		8 (2.5)	
	Other	3 (1)		2 (0.6)	
	Missing	0 (0)		2 (0.6)	
**Working experience (years)**					
	<5	48 (15.4)		83 (25.5)	
	6-10	76 (24.4)		48 (14.7)	
	11-15	53 (17)		67 (20.6)	
	16-20	55 (17.7)		40 (12.3)	
	>20	79 (25.4)		87 (26.7)	
	Missing	0 (0)		1 (0.3)	
**Education or training**					
	<1 year	79 (25.4)		84 (25.8)	
	>1 year	120 (38.6)		113 (34.7)	
**Organizational policy toward eHealth use**					
	Voluntary	232 (74.6)		223 (68.4)	
	Required	52 (16.7)		54 (16.6)	
	Organization did not use eHealth	26 (8.4)		13 (4)	
	Missing	1 (0.3)		36 (11)	

^a^Sum of 2 types of residential care.

### Procedure

Participants were recruited from 5 health care organizations for people with ID, located in both urban and rural areas in the western and southern regions of the Netherlands. In both 2018 and 2021, professionals were invited to participate by personalized emails sent either by the researchers or a designated contact person from the participating organization. The email addresses were obtained from human resources employees with the approval of the board of directors of the care organization. The email invitation included a link to the web-based questionnaire, which was constructed using the Qualtrics software program, as well as an information sheet about the study. In 2021, one organization preferred an indirect invitation approach by placing the survey link and information sheet on their internal organizational website. In both years, a reminder was sent to participants within a month of the initial invitation. The 2018 survey was open for responses from December 2017 to April 2018. In 2021, the survey remained open from June to September.

Participants provided electronic informed consent after reviewing the information about their rights, data protection, and processing of data provided in the web-based questionnaire. The survey was designed to maintain anonymity, ensuring the confidentiality of participants’ responses.

### Measures

#### Acceptance and Use of eHealth

For this study, the authors adapted and extended the UTAUT questionnaire [19] for health care professionals working in care organizations for people with ID. This process involved 5 steps. In the first step, 2 focus groups with health care professionals working with people with ID and familiar with eHealth discussed the suitability of the UTAUT model factors and the corresponding 19 items in the context of ID. Based on their feedback, 1 original facilitating conditions item did not fit the present context (“If I use the system, I will increase my chances of getting a raise”), and 6 additional items were added to enhance alignment with the work context; that is, 3 items were added to the performance expectancy factor, focusing on collaboration, working together with clients, and effectiveness of support or therapy provision. Furthermore, 3 items were also added to the facilitating conditions factor, addressing the client’s facilities, digital literacy, and health care professionals’ time availability. In the second step, the original English items were translated into Dutch using a stepwise forward-backward translation procedure [31]; that is, the original English items were translated into Dutch by 2 researchers independently, then back-translated into English by 2 native speakers. Third, a consensus Dutch translation was achieved by the 2 researchers with the help of an experienced manager familiar with health care organizations for people with ID, resulting in minor revisions for item clarification. The fourth step involved 3 health care professionals reviewing the adapted items to assess their suitability for various eHealth tools, such as videoconferencing and virtual reality. Minimal adjustments were made to the item formulation based on their feedback. Finally, in the fifth step, the wording and sequence of the survey items were reviewed, and a pilot survey flow was tested by 6 fellow researchers. Minor suggestions from this pilot testing were incorporated into the final survey. These 5 steps resulted in an extended UTAUT survey consisting of 25 items, all measured on a 5-point Likert scale response format ranging from 1 (totally disagree) to 5 (totally agree). Higher scores indicated a greater acceptance of using eHealth to support or provide therapy for people with ID.

In addition to the UTAUT-based questionnaire, information on eHealth usage by health care professionals was collected. Questions assessed familiarity, frequency, and intensity of usage for 6 eHealth applications: apps, eHealth platforms, serious gaming, videoconferencing, video modeling, and virtual reality. These eHealth tools were selected based on existing literature and their relevance to clinical practice for people with ID [32-34].

#### eHealth Experience and Voluntariness

To measure eHealth experience, which was a moderator in this study, the computer self-efficacy subscale of the Dutch e-Health attitude questionnaire [35] was used. This subscale consisted of 7 items that evaluated personal experience with information and communication technology (eg, “I feel capable of using various computer programs”). In addition, 3 items were reverse-coded, and participants responded on a 5-point Likert scale ranging from 1 (totally disagree) to 5 (totally agree). Higher scores indicated greater experience with using IT and computer programs.

To assess the voluntariness of eHealth use, which was also a moderator in this study, a single item inquiring about the organization’s policy on eHealth usage (“How is eHealth usage arranged within your organization?”) was included in the survey.

#### Digital Working Alliance

In order to explore participants’ perception of the contribution of eHealth to the working alliance and its impact on their intention to use eHealth, a digital working alliance was included as a moderator in 2021. To measure participants’ perception of the digital working alliance, the web-based questionnaire included an adapted Technical Alliance Inventory (TAI). Participants working with people with mild ID completed the TAI–Short Form–Mild ID (11 items) [36], while those working with people with severe ID completed the TAI–Short Form–Severe ID (12 items). The items were rated on a 5-point Likert scale, ranging from 1 (totally disagree) to 5 (totally agree). Higher scores indicated a more positive perception of the role of eHealth in collaboration with people with ID. Further details on the psychometric properties of the TAI–Short Form–Mild ID for professionals working with mild ID can be found in the study of Oudshoorn et al [36].

#### eHealth Training

To enhance the understanding of the organizational context as perceived by participants, 2 additional items were included in the questionnaire to assess the training they received in working with eHealth.

#### Impact of COVID-19 Pandemic

The impact of the COVID-19 pandemic on the acceptance and usage of eHealth by health care professionals was captured by 3 items based on relevant literature [37-39]: “Due to the COVID-19 pandemic I have used eHealth increasingly,” “Due to the Covid-19 pandemic I have used eHealth differently,” and “Due to the Covid-19 pandemic I have a different perception of eHealth use.” Participants rated these items on a 5-point Likert scale ranging from 1 (totally disagree) to 5 (totally agree). Higher scores indicated a greater impact of the pandemic on eHealth acceptance and use. In 2021, the sequence of items was adjusted to ensure a logical flow of the survey in light of the inclusion of additional topics.

#### Demographic Information

Gender and age, 2 moderators in this study, were measured as part of the demographic information collected, which also included profession, working experience, and educational level. Age was categorized into 4 groups: <30, 30-39, 40-49, and ≥50 years. Gender was measured by male, female, or other.

### Analysis

The data analysis was conducted using Mplus (version 8.1; Muthén & Muthén) [40] and IBM SPSS for Windows (version 25). Only participants who filled out ≥80% (≥20/25) of the UTAUT statements were included in further data analysis. The analysis involved 5 steps to examine the factors and relationships within the dataset. First, a combination of confirmatory factor analysis (CFA) and exploratory factor analysis (EFA) was used to evaluate the factor structure for the UTAUT model among health care professionals in support and psychological therapy for people with ID, following the approach of Békés et al [23]. The original 4-factor UTAUT model, as well as the extended 4-factor model with 6 additional items, were tested using CFA. The EFA aimed to identify latent constructs and to arrive at a parsimonious representation of the associations among measured variables. Data from the 2018 dataset were used for these analyses. With respect to the CFA, the maximum likelihood with robust SEs estimator for continuous data was used, treating the 5-point Likert scale responses as continuous given the adequacy of the continuous maximum likelihood with robust SEs estimator for ordinal data with ≥5 categories [41]. Several fit statistics were used to examine goodness-of-fit, with acceptable model fit indicated by normed *ꭓ*^2^ <3.00, root mean square error of approximation (RMSEA) <0.08, comparative fit index (CFI) >0.90, and standardized root mean square residual (SRMR) <0.10 [42,43].

Second, because the CFA did not yield a satisfactory model fit (additional information in the *Results* section), an EFA was deployed to explore the factor structure based on the procedure described in the development of the UTAUT-Therapist (UTAUT-T) model by Békés et al [23]. Bartlett test of sphericity was significant (*χ*^2^_210_=3133.886, *P*<.001), indicating that it was appropriate to use the factor analytic model on this dataset. Next, the Kaiser-Meyer-Olkin (KMO) measure of sampling adequacy indicated that the strength of the variables’ relationship was high (KMO=0.86), justifying the execution of EFA. The 25 items were subjected to maximum likelihood factor extraction with Oblimin rotation. Based on the commonly accepted extraction rules (scree plots, eigenvalues>1, items with factor loadings >.40), 5 factors were found.

Third, descriptive statistics were calculated for both datasets to provide an overview of the data. Fourth, a multiple regression analysis was performed to examine the impact of the 5 individual factors on the Behavioral intention factor. Finally, a stepwise regression analysis was conducted to explore the potential role of 4 moderators (gender, age, experience, and voluntariness of eHealth use) on the relationship between the 5 UTAUT factors and Behavioral intention. In the regression analysis for 2021, the technical alliance mean score was included as a fifth moderator. No Bonferroni corrections were applied to the separate regression analyses due to the study’s exploratory nature and focus on individual scores of the 5 factors [44].

### Ethical Considerations

The Ethics Review Board of Tilburg University approved the study (EC-2016.71). Participants were informed of the study’s purpose and their rights on the front page of the web-based questionnaire, and they were required to provide consent before accessing the questionnaire. At the end of the questionnaire, participants were given the option to provide their email addresses if they wished to receive updates about the study’s findings. Any email addresses and other personal information, including automatically collected IP addresses from Qualtrics, were removed from the data file prior to analysis. All participants were employed by the care organizations involved in the Academic Collaborative Centre Living with an Intellectual Disability (Tranzo, Tilburg University). They volunteered to participate and did not receive any compensation or gifts for their involvement.

## Results

To answer the first research question on the applicability of the UTAUT model, a CFA followed by an EFA were conducted to determine model fit.

### CFA and Extended UTAUT Model

The original 4-factor model and the extended 4-factor model, including 6 additional items, exhibited inadequate model fit in the CFA. Specifically, the original 4-factor model had a normed *χ^2^* of 3.25, RMSEA=0.085, CFI=0.868, and SRMSR =0.109, while the extended 4-factor model had a normed *χ^2^* of 3.09, RMSEA=0.082, CFI=0.832, and SRMSR=0.103. These findings suggest that neither of the UTAUT models was suitable for the present dataset.

### EFA Results

EFA was used to explore a new model. Table 2 presents the pattern matrix obtained, including only items with factor loadings >0.40 (1 item excluded from the dataset). The pattern matrix revealed the presence of 5 factors: Factor 1, Perceived added value (7 items; α=.85); Factor 2, Convenience and self-confidence (6 items; α=.78); Factor 3, Social pressure from colleagues and support from manager (3 items, α=.79); Factor 4, Organizational support (3 items, α=.76); and Factor 5, Facilitating conditions for people with ID (such as devices and digital skills; 2 items, α=.78). In addition, 3 items composed the Behavioral intention factor (3 items, α=.95).

**Table 2:** Factor loadings of Unified Theory of Acceptance and Use of Technology (UTAUT) items and added items from the focus group consultation.

Item	Factor 1	Factor 2	Factor 3	Factor 4	Factor 5
Using eHealth facilitates working together with my client to reach their goals.^a^	0.703	–0.008	0.068	0.108	0.171
The use of eHealth supports the provision of support/therapy more effectively.^a^	0.697	0.207	0.000	–0.076	0.004
eHealth enables collaboration with other persons involved in the client’s formal and informal network.^a^	0.693	0.051	–0.037	0.130	–0.084
I find eHealth useful for my work.	0.687	0.150	–0.016	0.160	–0.011
It would be easy for me to become skillful in using eHealth.	0.638	–0.192	0.034	0.045	0.089
Using eHealth increases my productivity.	0,554	0.407	0.057	–0.378	–0.037
Using eHealth enables me to accomplish tasks more quickly.	0.541	0.373	0.157	–0.305	–0.083
I clearly understand how to use eHealth as part of the support and/or therapy I provide	–0.007	0.723	–0.050	0.168	0.075
I have the knowledge necessary to use eHealth.	–0.189	0.709	0.036	0.308	0.147
I find eHealth easy to use.	0.199	0.698	–0.053	0.013	0.038
By using eHealth, I will increase the extent to which I am valued (eg, I am able to get a targeted training, I could become an eHealth ambassador in my organization).^b^	0.227	0.610	0.064	–0.369	–0.160
Learning to operate an eHealth tool is easy for me.	0.087	0.533	0.011	0.068	0.105
I have sufficient time to make eHealth my own.^a^	–0.040	0.533	0.161	0.195	0.198
Colleagues who influence my behavior think that I should use eHealth.	–0.068	–0.051	0.980	–0.034	–0.038
Colleagues who are important to me think that I should use eHealth.	0.000	–0.095	0.972	–0.021	0.006
The senior management of my care organization has been helpful in the use of eHealth.	0.125	0.222	0.430	0.395	0.046
There is a specific person (or group) available for assistance with eHealth difficulties.	0.140	0.037	–0.007	0.798	–0.045
In general, the organization has supported the use of eHealth.	0.293	0.095	0.063	0.727	0.010
I have the resources necessary to use eHealth.	–0.062	0.298	0.056	0.613	0.083
My client has the facilities (eg, computer, laptop, smartphone, internet access) necessary to use eHealth.^a^	0.025	0.073	–0.030	–0.092	0.902
My client has the necessary digital literacy to use eHealth.^a^	0.069	0.006	0.009	–0.075	0.901

^a^Added items by focus group consultation.

^b^Original UTAUT item adapted for cross-cultural reasons; one item with factor loading <.40 deleted

To address the second research question on the level of acceptance and use of eHealth among health care professionals working with individuals with intellectual disabilities, descriptive analysis, regression analysis, and moderator analysis were conducted. The results of these analyses are presented sequentially.

### Descriptive Analysis

Tables 3 and 4 present the descriptive statistics and correlations of the 5 factors in the extended UTAUT model derived from both datasets. Mean scores were calculated for each factor to assess the acceptance of eHealth among support staff and therapists. Acceptance scores were categorized as low (1-2.34), moderate (2.35-3.67), or high (3.68-5) following the acceptance study by Henneman et al [17]. In both the 2018 and 2021 datasets, the mean scores for all 5 factors were found to be moderate. For more detailed information, refer to Tables 3 and 4. Item mean and SD scores can be found in Multimedia Appendix 1. In addition, participants in 2021 expressed agreement that the COVID-19 pandemic had resulted in increased eHealth usage (mean 3.85, SD 1.008). They also indicated that their use of eHealth changed due to the pandemic (mean 3.58, SD 1.054) and that it had influenced their opinion about eHealth (mean 3.58, SD 1.008).

**Table 3:** Mean and SD of factors and intercorrelations, 2018 dataset.

Factor			Score, mean (SD)		Factor 1		Factor 2		Factor 3		Factor 4		Factor 5		Behavioral intention
**1. Perceived added value**															
	*r*	3.46 (0.606)		1		.654		.320		.264		.187		.436	
	*P* value	—^a^		—		<.001		<.001		<.001		<.001		<.001	
**2. Convenience and self-confidence**															
	*r*	3.18 (0.627)		.654		1		.355		.503		.354		.548	
	*P* value	—		<.001		—		1		<.001		<.001		<.001	
**3. Social pressure from colleagues and support from manager**															
	*r*	2.64 (0.811)		.320		.355		1		.503		.354		.548	
	*P* value	—		<.001		<.001		—		<.001		<.001		<.001	
**4. Organizational support**															
	*r*	3.41 (0.824)		.264		.503		.352		1		.310		.581	
	*P* value	—		<.001		<.001		<.001		—		<.001		<.001	
**5. Facilitating conditions of client with intellectual disabilities**															
	*r*	2.84 (0.898)		.187		.354		.181		.310		1		.400	
	*P* value	—		<.001		<.001		<.001		<.001		—		<.001	
**Behavioral intention**															
	*r*	3.66 (0.883)		.436		.548		.298		.581		.400		1	
	*P* value	—		<.001		<.001		<.001		<.001		<.001		—	

^a^Not applicable.

**Table 4:** Mean and SD of factors and intercorrelations, 2021 dataset.

Factor			Score, mean (SD)		Factor 1		Factor 2		Factor 3		Factor 4		Factor 5		Behavioral intention
**1. Perceived added value**															
	*r*	3.44 (0.580)		1		.653		.260		.341		.329		.558	
	*P* value	—^a^		—		<.001		<.001		<.001		<.001		<.001	
**2. Convenience and self-confidence**															
	*r*	3.18 (0.591)		.653		1		.369		.585		.398		.508	
	*P* value	—		<.001		—		<.001		<.001		<.001		<.001	
**3. Social pressure from colleagues and support from manager**															
	*r*	2.57 (0.797)		.260		.369		1		.403		.283		.314	
	*P* value	—		<.001		<.001		—		<.001		<.001		<.001	
**4. Organizational support**															
	*r*	3.27 (0.845)		.341		.585		.403		1		.281		.511	
	*P* value	—		<.001		<.001		<.001		—		<.001		<.001	
**5. Facilitating conditions of client with intellectual disabilities**															
	*r*	2.63 (0.885)		.329		.398		.283		.281		1		.312	
	*P* value	—		<.001		<.001		<.001		<.001		—		<.001	
**Behavioral intention**															
	*r*	3.71 (0.872)		.558		.508		.314		.511		.312		1	
	*P* value	—		<.001		<.001		<.001		<.001		<.001		—	

^a^Not applicable.

Regarding the moderator experience, participants in both 2018 and 2021 reported high levels, with mean scores of 3.79 (SD 0.67) and 3.73 (SD 0.70), respectively. Table 5 presents the descriptive statistics for familiarity and actual usage of different eHealth tools. In 2018, support staff and therapists were most familiar with apps and virtual reality; 73% (213/311) of participants reported using apps, and 37% (65/311) reported using video modeling. Both apps and video modeling were primarily used in support and therapy for over a year.

**Table 5:** Familiarity and actual use of 6 eHealth applications of participants in 2018 and 2021.

Application			2018 survey (n=311)		2021 survey (n=326)
**Apps**					
	Familiarity, n (%)	292 (93.9)		291 (89.3)	
	Actual use^a^, n (%)	213 (72.9)		216 (74.2)	
	Use since (<1 y), n	76		54	
	Use since (>1 y), n	137		161	
	Use frequency (<1/wk^b^), n	61		60	
	Use frequency (≥1/wk^c^), n	152		156	
**Videoconferencing**					
	Familiarity, n (%)	145 (46.6)		315 (96.6)	
	Actual use, n (%)	57 (39.3)		218 (69.2)	
	Use since (<1 y), n	30		76	
	Use since (>1 y), n	27		142	
	Use frequency (<1/wk), n	27		106	
	Use frequency (≥1/wk), n	30		112	
**e-Health platform**					
	Familiarity, n (%)	201 (64.6)		126 (38.7)	
	Actual use, n (%)	116 (57.7)		49 (38.9)	
	Use since (<1 y), n	69		14	
	Use since (>1 y), n	47		35	
	Use frequency (<1/wk), n	51		28	
	Use frequency (≥1/wk), n	65		21	
**Virtual reality**					
	Familiarity, n (%)	222 (71.6)		221 (67.8)	
	Actual use, n (%)	19 (8.6)		15 (6.8)	
	Use since (<1 y), n	11		6	
	Use since (>1 y), n	8		9	
	Use frequency (<1/wk), n	16		12	
	Use frequency (≥1/wk), n	3		3	
**Serious gaming**					
	Familiarity, n (%)	53 (17)		49 (15)	
	Actual use, n (%)	4 (7.5)		7 (14.3)	
	Use since (<1 y), n	2		2	
	Use since (>1 y), n	2		5	
	Use frequency (<1/wk), n	3		6	
	Use frequency (≥1/wk), n	1		1	
**Video modeling**					
	Familiarity, n (%)	174 (55.9)		197 (60.4)	
	Actual use, n (%)	65 (37.4)		126 (64)	
	Use since (<1 y), n	26		42	
	Use since (>1 y), n	39		82	
	Use frequency (<1/wk), n	57		90	
	Use frequency (≥1/wk), n	8		36	

^a^The denominator is the n value in the “Familiarity” row.

^b^≤1/wk: once a month and incidental use were added.

^c^≥1/wk: daily, 2-3 times, and once a week were added.

In 2021, the majority of participants were familiar with videoconferencing (315/326, 96.6%), apps (291/326, 89.3%), and virtual reality (221/326, 67.8%). Specifically, 74.2% (216/291) of participants reported using apps, 69.2% (218/315) reported using videoconferencing, and 64% (126/197) reported using video modeling. It is worth noting that the adoption of videoconferencing may have been more recent, potentially influenced by the ongoing COVID-19 pandemic during the data collection period.

### Multiple Regression Analysis

A multiple regression analysis was conducted to examine the effect of the 5 factors on behavioral intention. In 2018, the combined influence of the 5 factors significantly predicted behavioral intention (*R*^2^=.47; *F*_5,305_=54.89, *P*<.001). In total, 4 factors had individual significant effects on behavioral intention: Factor 1 (β=.19; *t*=3.46; *P*=.001), Factor 2 (β=.16; *t*=2.46; *P*=.02), Factor 4 (β=.39; *t*=7.78; *P*<.001), and Factor 5 (β=.19; *t*=4.13; *P*<.001). However, Factor 3 did not show a significant effect on behavioral intention (β=.01; *t*=0.215; *P*=.83).

In 2021, the combined influence of the 5 factors also predicted behavioral intention significantly (*R*^2^=0.43; *F*_5,320_=49.32, *P*<.001). In addition, 2 factors had individual significant effects on behavioral intention: Factor 1 (β=.41; *t*=7.28; *P*<.001) and Factor 4 (β=.33; *t*=6.15; *P*<.001). Factor 2 (β=.001; *t*=0.019; *P*=.99), Factor 3 (β=.06; *t*=1.160; *P*=.25), and Factor 5 (β=.07; *t*=1.49; *P*=.1) had no significant effect on behavioral intention.

### Moderator Analyses

Finally, we conducted moderation analyses to examine the potential moderating effects of age, gender, experience, voluntariness, and technical alliance on the relationship between the individual 5 UTAUT factors measuring the construct acceptance and behavioral intention. Only the significant moderating effects are reported here; detailed results for all moderation analyses in the 2018 and 2021 datasets can be found in Multimedia Appendix 2.

In the 2018 dataset, several significant moderating effects were observed. In 3 different age groups, the relationship between individual UTAUT factors and intention to use eHealth was moderated differently. First, age was found to moderate the relationship between Factor 1 and Behavioral intention negatively for the 40-49 years age group (β=–.357; *P*=.04). This suggests that this age group was not as motivated to engage in the intended behavior when perceiving less added value. Second, for the same age group, the relationship between Factor 4 and Behavioral intention was again negatively moderated (β=–.273; *P*=.03), indicating that they perceived less organizational support for their intended behavior. Third, age also significantly moderated the relationship between Factor 3 and Behavioral intention; that is, a negative relationship was found for the 30-39 years age group (β=–.281; *P*=.02), while a positive relationship was observed for the ≥50 years age group (β=.332; *P*=.04). This suggests that the intended behavior of younger professionals (aged 30-39 years) was less influenced by colleagues and their managers, whereas for professionals aged ≥50 years, the opposite held true.

Experience as a moderator had a negative effect on the relationship between Factor 4 and Behavioral intention (β=–.167; *P*=.03), indicating that those with more experience may be less motivated to engage in the intended behavior when perceiving less organizational support. Last, voluntariness as a moderator had a negative effect on the relationship between Factor 5 and Behavioral intention (β=–.327; *P*=.004). This suggests that when the intended behavior is perceived as voluntary, the presence of facilitating conditions for people with ID may not be sufficient to motivate individuals to engage in the behavior.

In the 2021 dataset, gender was found to have a positive moderating effect on the relationship between Factor 2 and Behavioral intention (β=.376; *P*=.04), indicating that men felt more convenience and self-confidence to engage in the intended behavior. Gender also has a positive moderating effect on the relationship between Factor 4 and Behavioral intention (β=.341; *P*=.02), suggesting that men perceived more organizational support for their intended behavior. Moderator voluntariness had a negative effect on the relationship between Factor 3 and Behavioral intention (β=–.277; *P*=.02). Voluntariness also negatively moderated the relationship between Factor 4 and Behavioral intention (β=–.382; *P<*.001) as well as between Factor 5 and Behavioral intention (β=–.404; *P<*.001). These findings indicate that those who perceived the intended behavior as voluntary were less influenced by pressure from colleagues, support from their manager or the organization, or digital facilitating conditions for people with ID. Technical alliance moderated the relationship between Factor 5 and Behavioral intention positively (β=.157; *P=*.048). This suggests that when there is a higher level of technical alliance among support staff and therapists, the facilitating conditions for people with mild ID are perceived to be more effective in promoting behavioral intention.

## Discussion

### Principal Findings

This study aimed to assess the applicability of the UTAUT model in understanding health care professionals’ acceptance and intention to use eHealth for support and psychological therapy among people with ID. In addition, it explored the level of acceptance and use of eHealth among support staff and therapists providing support and psychological therapy for people with ID and whether the acceptance and usage changed during the COVID-19 pandemic.

With respect to the first research question (applicability of the UTAUT model), a questionnaire based on the UTAUT model was adapted and extended for health care professionals working with people with ID. However, based on this questionnaire, neither the original UTAUT model nor the extended UTAUT model yielded satisfactory model fit results according to the CFA. Therefore, an EFA was conducted to explore the underlying latent factors for the extended model, resulting in a 5-factor model demonstrating acceptable-to-good internal consistency. This extended model served as the reference for further analysis of the acceptance of eHealth among support staff and therapists in 2 cross-sectional, web-based questionnaire studies in 2018 and 2021. The 5-factor model, which determined acceptance, accounted for 43% to 47% of the variance in the intention to use eHealth. This is in line with the findings of the UTAUT-T [23], showing that the 5 UTAUT-T subscales (Therapy quality expectancy, Pressure from others, Professional support, Ease of use, and Convenience) collectively predicted 42% of the average behavioral intention.

Other studies applying the UTAUT model to investigate acceptance among health care professionals working with a general patient or client populations found varied explained variance in intended behavior percentages, ranging from 31% to 78% [45]. This led to the conclusion that the extended UTAUT model partially applies to understanding the acceptance and intention to use eHealth of support staff and therapists working with people with ID. The factor “Facilitating conditions of clients,” which was included in this study based on the advice of the expert group, was confirmed in factor analysis as a relevant factor for acceptance. Notably, this unique factor was not present in previous health care context studies examining the UTAUT model or recommended for inclusion in future studies [17,23,46]. Furthermore, CFA is needed to examine the extended UTAUT model among a larger group of health care professionals working with people with ID in order to establish the generalizability and robustness of the extended UTAUT model’s findings.

While the extended UTAUT model partially applies to eHealth acceptance and intention among support staff and therapists working with individuals with ID, there are still unknown factors influencing their intentions to use eHealth. A possible reason for this knowledge gap is that the UTAUT model primarily focuses on individual user perspectives at specific moments, overlooking contextual factors that affect eHealth implementation and the roles of health care professionals [18,47]. This one-sided perspective of the UTAUT model might not align well with multilateral contexts within health care organizations, which significantly influence health care professionals’ behavior [48,49]. The 5-factor UTAUT model developed in this study can be applied to understand the individual factors that affect support staff and therapists’ intentions to use eHealth in providing support and therapy among people with intellectual disabilities. However, fully understanding professionals’ motivation requires supplementing this model with a focus on contextual factors captured in other theories and models (eg, the Nonadoption, Abandonment, Scale-Up, Spread, and Sustainability framework by Greenhalgh et al [50]).

With regard to the second research question (level of acceptance and use of eHealth), this study found that support staff and therapists demonstrated moderate acceptance, determined by 5 influencing factors. Notably, the perceived added value of eHealth and organizational support emerged as the primary drivers for acceptance, consistent with previous research [45,51]. Interestingly, participants in our study did not experience significant social pressure from colleagues to adopt eHealth, aligning with findings in rehabilitation care [21], primary mental health care [22] and psychotherapy [52]. Instead, their willingness to use eHealth appeared to be more dependent on perceived benefits for their clients [53,54]. However, in this study, participants expressed concerns regarding the facilitating conditions for clients, such as access to proper equipment and digital skills required to benefit from eHealth interventions, a sentiment echoed in several studies [55,56]. Furthermore, the study’s moderator analysis revealed that participants aged 40-49 years, as well as those who viewed the use of eHealth as voluntary, displayed a negative influence on their intention to adopt eHealth. Chiu and Ku [57] state that factors influencing eHealth use might differ in health care organizations with mandatory or voluntary use policies. The role of age in eHealth adoption has been studied, but findings have not been consistent [51,58].

With regard to the eHealth usage, the following patterns were observed for 2018 and 2021. Participants in 2018 showed a preference for using apps and video modeling most frequently. In 2021, this trend continued, with apps and video modeling remaining the most commonly used eHealth tools. Notably, video modeling saw a more substantial increase in usage compared to 2018; telecare, particularly video conferencing technology, experienced a significant surge in adoption in 2021, likely attributable to the impact of the COVID-19 pandemic. In 2021, the familiarity and use of eHealth platforms decreased compared with 2018. However, the adoption of more innovative eHealth tools like virtual reality and serious gaming remained limited in clinical practice both in 2018 and 2021. The findings align with observations in other care domains, such as mental health care, where videoconferencing also increased during the pandemic, but innovative tools continued to be underused [59].

Despite the differences in participant groups and contexts between the 2 surveys (2018 and 2021) due to their cross-sectional designs and the impact of the pandemic, the results pertaining to acceptance factors were found to be comparable. Participants in 2021 acknowledged that the pandemic significantly influenced their views on eHealth, as indicated by the additional COVID-19 questions. Contrary to our expectations, this influence did not lead to a distinct acceptance profile based on the extended UTAUT model. The similarity in acceptance profiles observed in both survey years might be explained by several factors. First, the significant increase in videoconferencing in 2021 may have played a role. Studies show that videoconferencing can serve as a viable alternative to in-person services without negatively affecting acceptance [60]. Due to restrictive measures, outreach support staff and therapists had to adapt to virtual work, with videoconferencing proving to be a time-efficient alternative [61]. However, the main group of participants in this study consisted of residential support staff, who were less obligated to shift from face-to-face support to remote support, potentially influencing acceptance scores. Lastly, over time, the surveyed support staff and therapists may have become more accustomed to videoconferencing a year after the start of the pandemic, which could explain the consistent acceptance profile. In previous studies, findings on the impact of the COVID-19 pandemic on eHealth acceptance among health care professionals described some health care professionals reporting negative experiences, as well as others who felt surprised about the opportunities [62-64]. In this study, despite the pandemic’s influence, the acceptance profile remained comparable between the 2 survey years. The reasons for the consistent acceptance profiles of eHealth over time and the variables that contribute to these stable acceptance profiles require further investigation in the near future. Knowledge about eHealth is necessary for acceptance but insufficient for actual usage [65]. Factors like training, integrating eHealth into education, workflow, and organizational culture improve acceptance [51,64]. In our study, support staff and therapists lacked eHealth training, even during the pandemic. Accessible training methods, such as short videos demonstrating benefits, have proven effective [52]. However, research on eHealth acceptance and training needs of professionals working with people with ID is lacking and needs to build knowledge on facilitating conditions to ensure successful implementation. Conducting such research is crucial to adequately prepare professionals for effective eHealth use, enhancing the quality of care for this population. Further qualitative research on the experiences, motivations, and values of health care professionals using eHealth in care practice could provide a more comprehensive understanding of what drives their acceptance and how it impacts their collaboration with people with intellectual disabilities.

### Strengths and Limitations

The UTAUT model primarily focuses on the individual perspective of eHealth acceptance and usage, but these processes are complex and involve various factors [48]. In our study, we expanded the analysis to include organizational aspects like eHealth policy and training provided by care organizations. To gain a comprehensive understanding, future research should consider perspectives from people with ID and their relatives. Collaboration with professionals working with mild ID during survey development was a notable strength of this study.

Although we carefully translated UTAUT statements into Dutch, some items might not have accurately reflected participants’ clinical practice or their perception of “eHealth,” possibly impacting the model’s explained variance. Nevertheless, our study’s strengths include covering familiar eHealth tools and various working domains (community care and long-term care), representing a broad spectrum of professional care.

A potential limitation is self-selection bias, as those interested in eHealth and adept at web-based questionnaires might have been more likely to participate. Caution is needed when interpreting findings, avoiding automatic generalization to all support staff and therapists working with individuals with ID.

### Conclusion

In conclusion, the extended UTAUT model is partially applicable to understanding the acceptance and intention to use eHealth among health care professionals working with people with ID. Future research is needed to fully understand what additional factors determine health care professionals’ acceptance and eHealth use among clients with ID. The level of acceptance was moderate, with the perceived added value of using eHealth among clients with ID and organizational support as the most relevant determinants of acceptance. This study provides valuable insights into the acceptance of eHealth among support staff and therapists in health care organizations for people with ID, as they play a crucial role in supporting and motivating clients to embrace eHealth, making their acceptance relevant for the success of health care innovations [51].

## Appendix

Multimedia Appendix 1 Mean and SDs for factors and items of participants from 2018 and 2021.

Multimedia Appendix 2 Results of moderator analysis of data from 2018 and 2021.

## Abbreviations

CFA: confirmatory factor analysis
CFI: comparative fit index
EFA: exploratory factor analysis
ID: intellectual disabilities
KMO: Kaiser-Meyer-Olkin
RMSEA: root mean square error of approximation
SRMR: standardized root mean square residual
TAI: Technical Alliance Inventory
UTAUT: Unified Theory of Acceptance and Use of Technology
UTAUT-T: Unified Theory of Acceptance and Use of Technology-Therapist
