# Supplementary figures and images for: Acceptance and Use of eHealth in Support and Psychological Therapy for People With Intellectual Disabilities: Two Cross-Sectional Studies of Health Care Professionals
Source: JMIR Form Res. 2024 Nov 12;8:e52788. doi: 10.2196/52788 (PMC11599880; doi:10.2196/52788)

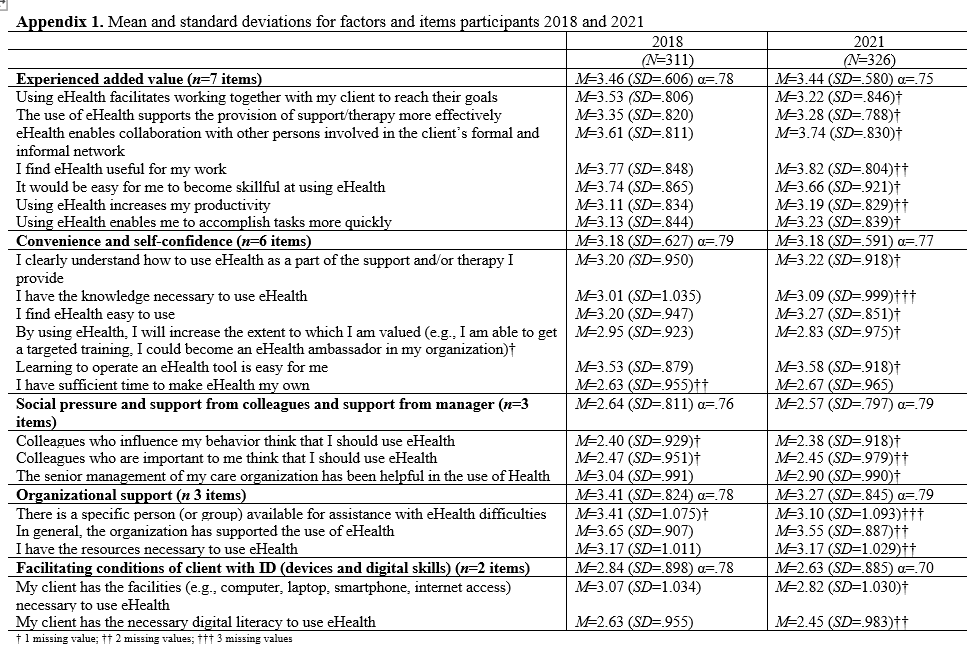

Supplement: Multimedia Appendix 1 [file formative_v8i1e52788_app1.docx]
