# Supplementary material for: Acceptance and Use of eHealth in Support and Psychological Therapy for People With Intellectual Disabilities: Two Cross-Sectional Studies of Health Care Professionals
Source: JMIR Form Res. 2024 Nov 12;8:e52788. doi: 10.2196/52788 (PMC11599880; doi:10.2196/52788)
Supplement: Multimedia Appendix 2 [file formative_v8i1e52788_app2.docx]

| **Appendix 2.** *Results of moderator analysis of data 2018 and 2021* | | | | | |  |
| --- | --- | --- | --- | --- | --- | --- |
| **2018** |  |  |  |  |  |  |
| **Moderator Gender on relationship Factors->BI** | | | | |  |  |
|  | B | SE | Beta | t | Sig |  |
| IntF1_Geslacht | -0,151 | 0,211 | -0,040 | -0,717 | 0,474 |  |
| IntF2_Geslacht | -0,328 | 0,211 | -0,079 | -1,556 | 0,121 |  |
| IntF3_Geslacht | -0,185 | 0,173 | -0,063 | -1,073 | 0,284 |  |
| IntF4_Geslacht | -0,333 | 0,175 | -0,092 | -1,908 | 0,057 |  |
| IntF5_Geslacht | -0,274 | 0,164 | -0,093 | -1,665 | 0,097 |  |
| **Dummy = Male** | | | | | |  |
|  |  |  |  |  |  |  |
| **Moderator Experience on relationship Factors-> BI** | | | | | |  |
|  | B | SE | Beta | t | Sig |  |
| IntF1_CompGeb | -0,072 | 0,100 | -0,036 | -0,721 | 0,471 |  |
| IntF2_CompGeb | -0,078 | 0,086 | -0,043 | -0,906 | 0,365 |  |
| IntF3_CompGeb | -0,118 | 0,090 | -0,068 | -1,318 | 0,188 |  |
| IntF4_CompGeb | -0,167 | 0,076 | -0,099 | -2,211 | 0,028 | p <.05 |
| IntF5_CompGeb | -0,046 | 0,071 | -0,032 | -0,646 | 0,519 |  |
|  |  |  |  |  |  |  |
| **Moderator Voluntariness on relationship Factors-> BI** | | | | | |  |
|  | B | SE | Beta | t | Sig |  |
| IntF1_Vrijblijv | -0,052 | 0,160 | -0,030 | -0,327 | 0,744 |  |
| IntF2_Vrijblijv | 0,014 | 0,143 | 0,008 | 0,098 | 0,922 |  |
| IntF3_Vrijblijv | -0,236 | 0,133 | -0,185 | -1,773 | 0,077 |  |
| IntF4_Vrijblijv | -0,106 | 0,106 | -0,081 | -1,001 | 0,318 |  |
| IntF5_Vrijblijv | -0,327 | 0,113 | -0,282 | -2,895 | 0,004 | p<.01 |
| **Dummy = Voluntariness** | | | | | |  |
|  |  |  |  |  |  |  |
| **Moderator Age ranges on relationship Factors-> BI 2018** | | | | | |  |
| **Age group < 30** | |  |  |  |  |  |
|  | B | SE | Beta | t | Sig |  |
| IntF1_LeeftijdCatJongerdan30 | 0,332 | 0,175 | 0,111 | 1,891 | 0,060 |  |
|  |  |  |  |  |  |  |
|  | B | SE | Beta | t | Sig |  |
| IntF2_LeeftijdCatJongerdan30 | 0,218 | 0,156 | 0,076 | 1,400 | 0,163 |  |
|  |  |  |  |  |  |  |
|  | B | SE | Beta | t | Sig |  |
| IntF3_LeeftijdCatJongerdan30 | 0,231 | 0,148 | 0,096 | 1,563 | 0,119 |  |
|  |  |  |  |  |  |  |
|  | B | SE | Beta | t | Sig |  |
| IntF4_LeeftijdCatJongerdan30 | 0,176 | 0,120 | 0,077 | 1,465 | 0,144 |  |
|  |  |  |  |  |  |  |
|  | B | SE | Beta | t | Sig |  |
| IntF5_LeeftijdCatJongerdan30 | 0,327 | 0,129 | 0,146 | 2,540 | 0,012 | p <.05 |
|  |  |  |  |  |  |  |
| **Age group 30-39** | |  |  |  |  |  |
|  | B | SE | Beta | t | Sig |  |
| IntF1_LeeftijdCat30_39 | 0,020 | 0,162 | 0,008 | 0,122 | 0,903 |  |
|  |  |  |  |  |  |  |
|  | B | SE | Beta | t | Sig |  |
| IntF2_LeeftijdCat30_39 | -0,179 | 0,141 | -0,076 | -1,272 | 0,204 |  |
|  |  |  |  |  |  |  |
|  | B | SE | Beta | t | Sig |  |
| IntF3_LeeftijdCat30_39 | -0,281 | 0,124 | -0,153 | -2,271 | 0,024 | p <.05 |
|  |  |  |  |  |  |  |
|  | B | SE | Beta | t | Sig |  |
| IntF4_LeeftijdCat30_39 | -0,127 | 0,103 | -0,073 | -1,241 | 0,215 |  |
|  |  |  |  |  |  |  |
|  | B | SE | Beta | t | Sig |  |
| IntF5_LeeftijdCat30_39 | -0,047 | 0,109 | -0,028 | -0,436 | 0,663 |  |
|  |  |  |  |  |  |  |
| **Age group 40-49** | |  |  |  |  |  |
|  | B | SE | Beta | t | Sig |  |
| IntF1_LeeftijdCat40_49 | -0,357 | 0,168 | -0,126 | -2,122 | 0,035 | p <.05 |
|  |  |  |  |  |  |  |
|  | B | SE | Beta | t | Sig |  |
| IntF2_LeeftijdCat40_49 | -0,226 | 0,172 | -0,069 | -1,312 | 0,190 |  |
|  |  |  |  |  |  |  |
|  | B | SE | Beta | t | Sig |  |
| IntF3_LeeftijdCat40_49 | -0,102 | 0,135 | -0,048 | -0,752 | 0,453 |  |
|  |  |  |  |  |  |  |
|  | B | SE | Beta | t | Sig |  |
| IntF4_LeeftijdCat40_49 | -0,273 | 0,126 | -0,111 | -2,167 | 0,031 | p <.05 |
|  |  |  |  |  |  |  |
|  | B | SE | Beta | t | Sig |  |
| IntF5_LeeftijdCat40_49 | -0,282 | 0,129 | -0,127 | -2,185 | 0,030 | p <.05 |
|  |  |  |  |  |  |  |
| **Age group > 50** | |  |  |  |  |  |
|  | B | SE | Beta | t | Sig |  |
| IntF1_LeeftijdCat50enouder | 0,033 | 0,193 | 0,010 | 0,169 | 0,866 |  |
|  |  |  |  |  |  |  |
|  | B | SE | Beta | t | Sig |  |
| IntF2_LeeftijdCat50enouder | 0,216 | 0,164 | 0,071 | 1,319 | 0,188 |  |
|  |  |  |  |  |  |  |
|  | B | SE | Beta | t | Sig |  |
| IntF3_LeeftijdCat50enouder | 0,332 | 0,157 | 0,129 | 2,109 | 0,036 | p <.05 |
|  |  |  |  |  |  |  |
|  | B | SE | Beta | t | Sig |  |
| IntF4_LeeftijdCat50enouder | 0,225 | 0,120 | 0,098 | 1,873 | 0,062 |  |
|  |  |  |  |  |  |  |
|  | B | SE | Beta | t | Sig |  |
| IntF5_LeeftijdCat50enouder | -0,007 | 0,116 | -0,004 | -0,061 | 0,951 |  |
|  |  |  |  |  |  |  |
|  |  |  |  |  |  |  |
| **2021** |  |  |  |  |  |  |
| **Moderator Gender on relationship Factors->BI** | | | | |  |  |
|  | B | SE | Beta | t | Sig |  |
| IntF1_Geslacht | 0,239 | 0,164 | 0,077 | 1,460 | 0,145 |  |
| IntF2_Geslacht | 0,376 | 0,181 | 0,112 | 2,080 | 0,038 | p <.05 |
| IntF3_Geslacht | 0,066 | 0,172 | 0,022 | 0,384 | 0,701 |  |
| IntF4_Geslacht | 0,341 | 0,149 | 0,120 | 2,293 | 0,022 | p <.05 |
| IntF5_Geslacht | 0,173 | 0,152 | 0,067 | 1,135 | 0,257 |  |
| **Dummy = Male** | | | | | |  |
| **Moderator Experience on relationship Factors-> BI** | | | | | |  |
|  | B | SE | Beta | t | Sig |  |
| IntF1_CompGeb | 0,099 | 0,081 | 0,059 | 1,219 | 0,224 |  |
| IntF2_CompGeb | 0,097 | 0,088 | 0,056 | 1,095 | 0,274 |  |
| IntF3_CompGeb | -0,022 | 0,075 | -0,016 | -0,299 | 0,765 |  |
| IntF4_CompGeb | 0,079 | 0,061 | 0,062 | 1,291 | 0,198 |  |
| IntF5_CompGeb | 0,050 | 0,068 | 0,040 | 0,737 | 0,461 |  |
|  |  |  |  |  |  |  |
| **Moderator Voluntariness on relationship Factors-> BI** | | | | | |  |
|  | B | SE | Beta | t | Sig |  |
| IntF1_Vrijblijv | -0,034 | 0,139 | -0,017 | -0,243 | 0,808 |  |
| IntF2_Vrijblijv | -0,212 | 0,137 | -0,104 | -1,550 | 0,122 |  |
| IntF3_Vrijblijv | -0,277 | 0,116 | -0,200 | -2,387 | 0,018 | p <.05 |
| IntF4_Vrijblijv | -0,382 | 0,097 | -0,289 | -3,930 | 0,000 | p <.001 |
| IntF5_Vrijblijv | -0,404 | 0,103 | -0,321 | -3,908 | 0,000 | p <.001 |
| **Dummy = Voluntariness** | | | | | |  |
|  |  |  |  |  |  |  |
| **Moderator Age ranges on relationship Factors-> BI 2021** | | | | | |  |
| **Age group < 30** | |  |  |  |  |  |
|  | B | SE | Beta | t | Sig |  |
| IntF1_LeeftijdCatJongerdan30 | 0,171 | 0,174 | 0,051 | 0,984 | 0,326 |  |
|  | B | SE | Beta | t | Sig |  |
| IntF2_LeeftijdCatJongerdan30 | 0,020 | 0,163 | 0,007 | 0,122 | 0,903 |  |
|  | B | SE | Beta | t | Sig |  |
| IntF3_LeeftijdCatJongerdan30 | -0,022 | 0,143 | -0,009 | -0,151 | 0,880 |  |
|  | B | SE | Beta | t | Sig |  |
| IntF4_LeeftijdCatJongerdan30 | 0,043 | 0,121 | 0,019 | 0,357 | 0,721 |  |
|  |  |  |  |  |  |  |
| IntF5_LeeftijdCatJongerdan30 | -0,146 | 0,132 | -0,065 | -1,106 | 0,270 |  |
|  |  |  |  |  |  |  |
| **Age group 30-39** | |  |  |  |  |  |
|  | B | SE | Beta | t | Sig |  |
| IntF1_LeeftijdCat30_39 | -0,034 | 0,144 | -0,014 | -0,239 | 0,811 |  |
|  | B | SE | Beta | t | Sig |  |
| IntF2_LeeftijdCat30_39 | 0,119 | 0,151 | 0,046 | 0,788 | 0,431 |  |
|  | B | SE | Beta | t | Sig |  |
| IntF3_LeeftijdCat30_39 | 0,110 | 0,120 | 0,061 | 0,920 | 0,358 |  |
|  | B | SE | Beta | t | Sig |  |
| IntF4_LeeftijdCat30_39 | 0,086 | 0,106 | 0,047 | 0,806 | 0,421 |  |
|  |  |  |  |  |  |  |
| IntF5_LeeftijdCat30_39 | 0,146 | 0,111 | 0,085 | 1,317 | 0,189 |  |
|  |  |  |  |  |  |  |
| **Age group 40-49** | |  |  |  |  |  |
|  | B | SE | Beta | t | Sig |  |
| IntF1_LeeftijdCat40_49 | 0,006 | 0,158 | 0,002 | 0,041 | 0,967 |  |
|  | B | SE | Beta | t | Sig |  |
| IntF2_LeeftijdCat40_49 | 0,041 | 0,174 | 0,013 | 0,237 | 0,813 |  |
|  | B | SE | Beta | t | Sig |  |
| IntF3_LeeftijdCat40_49 | 0,112 | 0,138 | 0,048 | 0,806 | 0,421 |  |
|  | B | SE | Beta | t | Sig |  |
| IntF4_LeeftijdCat40_49 | -0,072 | 0,116 | -0,035 | -0,623 | 0,534 |  |
|  |  |  |  |  |  |  |
| IntF5_LeeftijdCat40_49 | -0,033 | 0,123 | -0,016 | -0,271 | 0,786 |  |
|  |  |  |  |  |  |  |
| **Age group > 50** | |  |  |  |  |  |
|  | B | SE | Beta | t | Sig |  |
| IntF1_LeeftijdCat50enouder | -0,141 | 0,181 | -0,040 | -0,777 | 0,438 |  |
|  | B | SE | Beta | t | Sig |  |
| IntF2_LeeftijdCat50enouder | -0,185 | 0,173 | -0,057 | -1,068 | 0,286 |  |
|  | B | SE | Beta | t | Sig |  |
| IntF3_LeeftijdCat50enouder | -0,259 | 0,144 | -0,106 | -1,803 | 0,072 |  |
|  | B | SE | Beta | t | Sig |  |
| IntF4_LeeftijdCat50enouder | -0,077 | 0,122 | -0,034 | -0,629 | 0,530 |  |
|  |  |  |  |  |  |  |
| IntF5_LeeftijdCat50enouder | 0,017 | 0,123 | 0,008 | 0,138 | 0,890 |  |
|  |  |  |  |  |  |  |
|  |  |  |  |  |  |  |
| **2021** |  |  |  |  |  |  |
| **Moderator TAI MID on relationship Factors->BI** | | | | |  |  |
|  | B | SE | Beta | t | Sig |  |
| IntF1_TAIhoog | 0,160 | 0,121 | 0,103 | 1,326 | 0,187 |  |
| IntF2_TAIhoog | 0,084 | 0,116 | 0,056 | 0,727 | 0,469 |  |
| IntF3_TAIhoog | 0,084 | 0,116 | 0,056 | 0,727 | 0,469 |  |
| IntF4_TAIhoog | 0,118 | 0,086 | 0,103 | 1,368 | 0,174 |  |
| IntF5_TAIhoog | 0,157 | 0,079 | 0,150 | 1,993 | 0,048 |  |

*Note.* Results of moderator analysis with interaction between acceptance factors and behavioural intentions. All moderators belonging to the UTAUT model were tested. Moderator age was separated in four age groups: < 30, 30-39, 40-49, and > 50. Green marked boxes were positive moderators, orange marked boxes were negative moderators and yellow marked boxes were significant results.
